# Supplementary material for: Deconstructing Olfactory Epithelium Developmental Pathways in Olfactory Neuroblastoma
Source: Cancer Res Commun. 2023 Jun 6;3(6):980–90. doi: 10.1158/2767-9764.CRC-23-0013 (PMC10243222; doi:10.1158/2767-9764.CRC-23-0013)
Supplement: Table S1 — Tumor demographics separated by Hyams grade, Dulguerov T stage, and gene expression groups (low versus high) used for survival analysis in Figure 5 and Figure S4. Average Ki-67 mRNA expression obtained from bulk RNA-Seq outputs. Average Ki-67 index obtained from histology data reported by Classe et al., 2018. +/- in the last two columns is standard error of the mean (SEM). [file crc-23-0013-s01.docx]

|  | % high grade (III/IV) | % high T stage (3-4) | Avg. Ki-67 mRNA expression | Avg Ki-67 Index |
| --- | --- | --- | --- | --- |
| All Tumors | 52.63% | 73.68% | 1782.7 +/- 500.40 | 36.59 +/- 6.80 |
| Grade |  |  |  |  |
| Low grade (I/II) | 0.00% | 66.67% | 406.2 +/- 138.52 | 10.99 +/- 2.95 |
| High grade (III/IV) | 100.00% | 80.00% | 3021.6 +/- 759.06 | 57.08 +/- 6.85 |
| T Stage |  |  |  |  |
| Low stage (1-2) | 40.00% | 0.00% | 1417.21 +/- 990.55 | 39.93 +/- 21.29 |
| High stage (3-4) | 57.14% | 100.00% | 1913.24 +/- 598.10 | 35.64 +/- 6.90 |
| MKI67 Group |  |  |  |  |
| Low | 11.11% | 66.67% | 300.84 +/- 64.08 | 10.99 +/- 2.95 |
| High | 90.00% | 80.00% | 3116.38 +/- 728.70 | 57.08 +/- 6.85 |
| PLP1 Group |  |  |  |  |
| Low | 71.40% | 85.71% | 3184.74 +/- 1044.03 | 50.00 +/- 10.79 |
| High | 41.67% | 66.67% | 874.99 +/- 345.40 | 27.32 +/- 7.86 |
| S100B Group |  |  |  |  |
| Low | 62.50% | 87.50% | 2806.86 +/- 979.94 | 45.25 +/- 10.48 |
| High | 45.45% | 63.64% | 1037.86 +/- 397.34 | 29.67 +/- 8.77 |
| SOX9 Group |  |  |  |  |
| Low | 66.67% | 77.78% | 2634.56 +/- 903.85 | 44.26 +/- 10.24 |
| High | 40.00% | 70.00% | 1016.03 +/- 400.11 | 28.94 +/- 8.78 |
| TRPM5 Group |  |  |  |  |
| Low | 60.00% | 80.00% | 1905.24 +/- 704.75 | 37.36 +/- 8.75 |
| High | 44.44% | 66.67% | 1646.55 +/- 751.10 | 35.83 +/- 10.95 |

**Table S1:** Tumor demographics separated by Hyams grade, Dulguerov T stage, and gene expression groups (low versus high) used for survival analysis in Figure 5 and Figure S4. Average Ki-67 mRNA expression obtained from bulk RNA-Seq outputs. Average Ki-67 index obtained from histology data reported by Classe et al., 2018. +/- in the last two columns is standard error of the mean (SEM).
