# Supplementary figures and images for: Deconstructing Olfactory Epithelium Developmental Pathways in Olfactory Neuroblastoma
Source: Cancer Res Commun. 2023 Jun 6;3(6):980–90. doi: 10.1158/2767-9764.CRC-23-0013 (PMC10243222; doi:10.1158/2767-9764.CRC-23-0013)

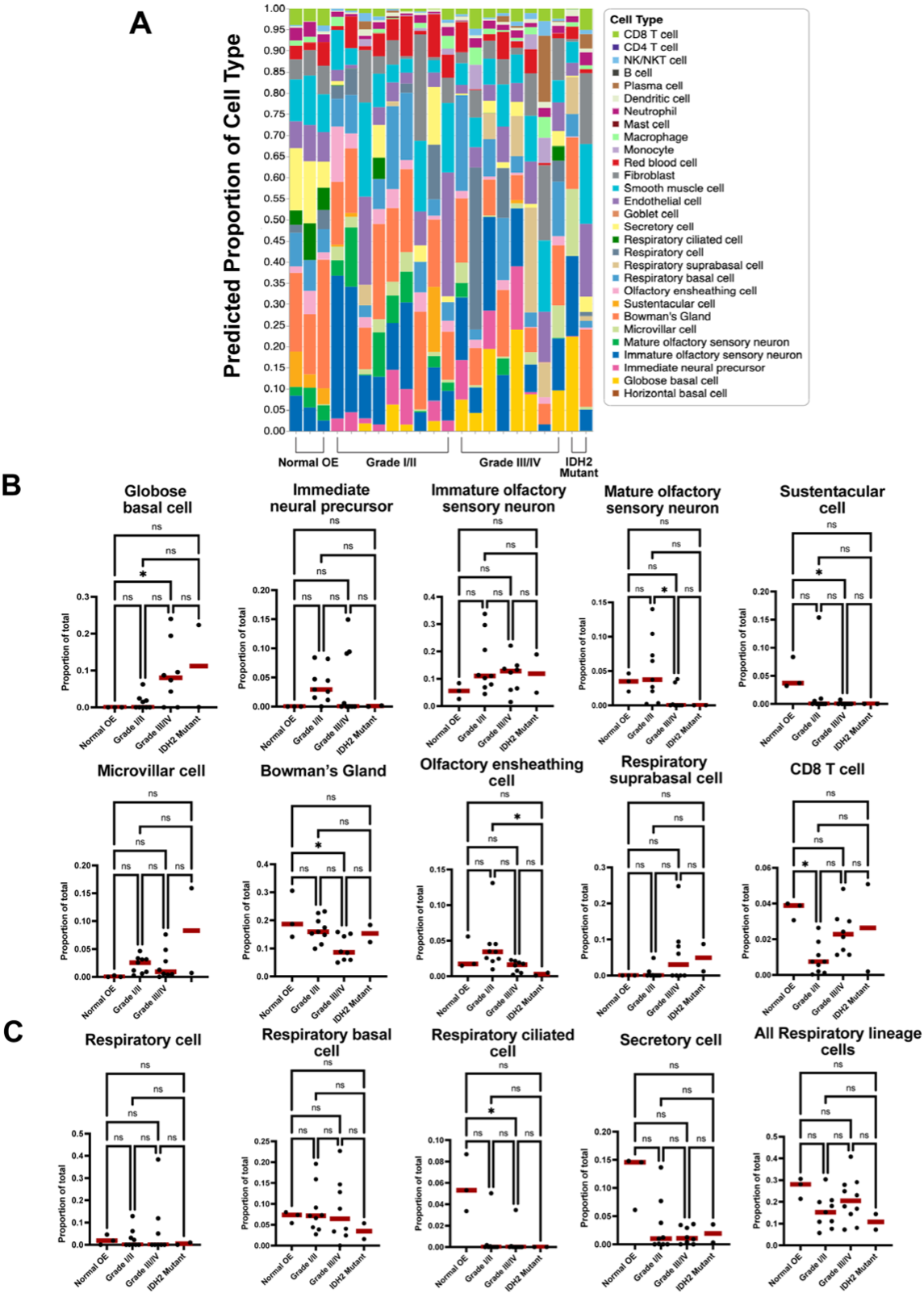

Supplement: Figure S1 — A and B identical to Figure 2A and B, but with the two IDH2 mutant tumors separated out from the other tumors into their own category. C shows estimated respiratory cell type proportions across normal OE, low grade, high grade, and IDH2 mutant tumors, which reveals that model estimates do not predict an increase in respiratory cell programming in IDH2 mutant tumors. The “All Respiratory lineage cells” plot includes cell type proportions from respiratory cells, respiratory basal cells, respiratory suprabasal cells, respiratory ciliated cells, and secretory cells. [file crc-23-0013-s02.png]

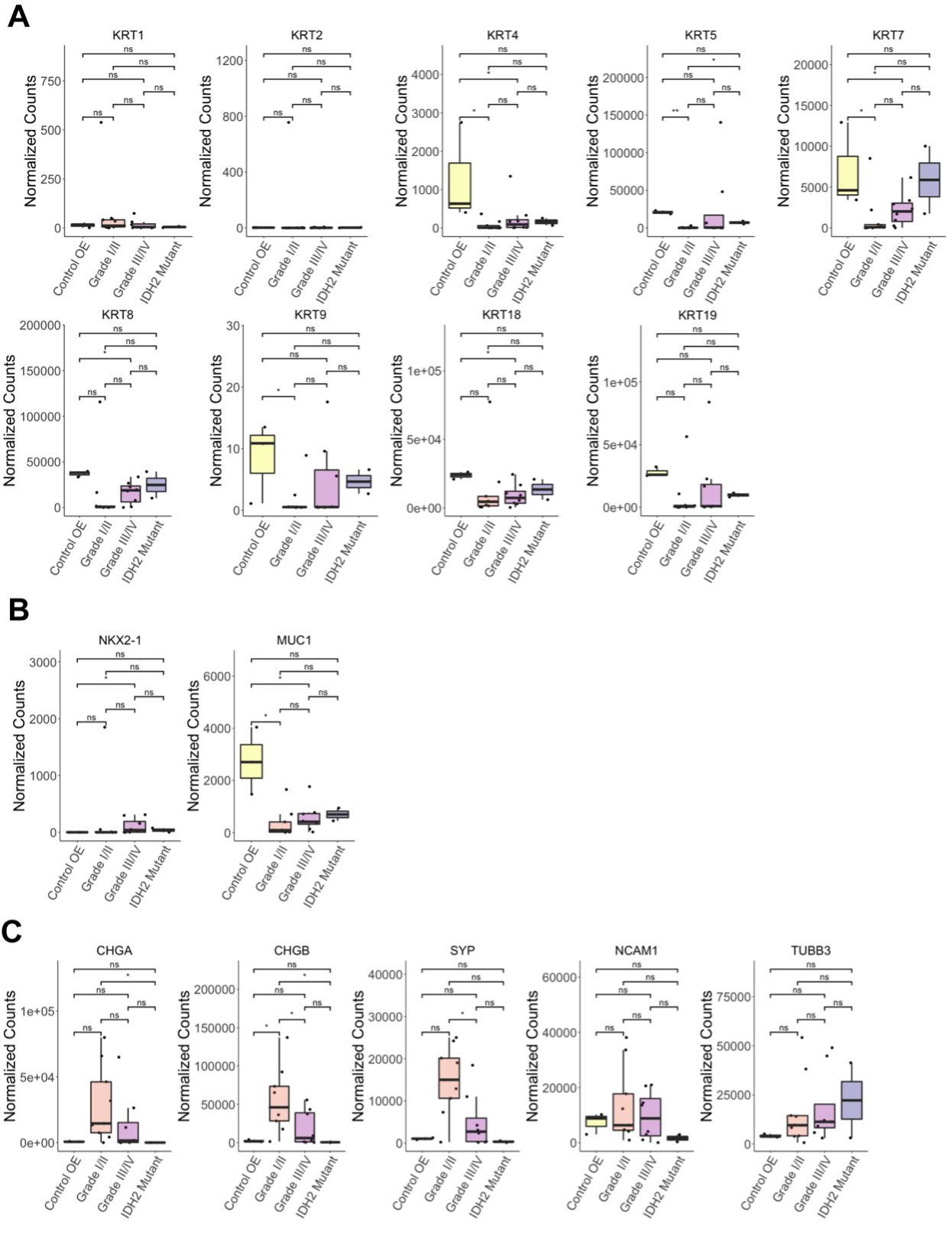

Supplement: Figure S2 — RNA expression signatures of ONB from Classe et al., 2018 based on markers traditionally associated with sinonasal undifferentiated carcinoma (A), small cell neuroendocrine carcinoma (B), and olfactory neuroblastoma (C), divided by control OE, low grade ONB, high grade ONB, and IDH2 mutant ONB. A) Expression of keratins is not significantly increased in IDH2 mutant ONB versus IDH2 wild type tumors. B) Small cell neuroendocrine carcinoma (SNEC) marker genes are not highly expressed ONB from Classe et al. dataset. C) Expression of canonical neuroendocrine markers is negatively associated with ONB grade, and is not significantly different between high grade IDH2 and high grade IDH2 mutant ONB. [file crc-23-0013-s03.png]

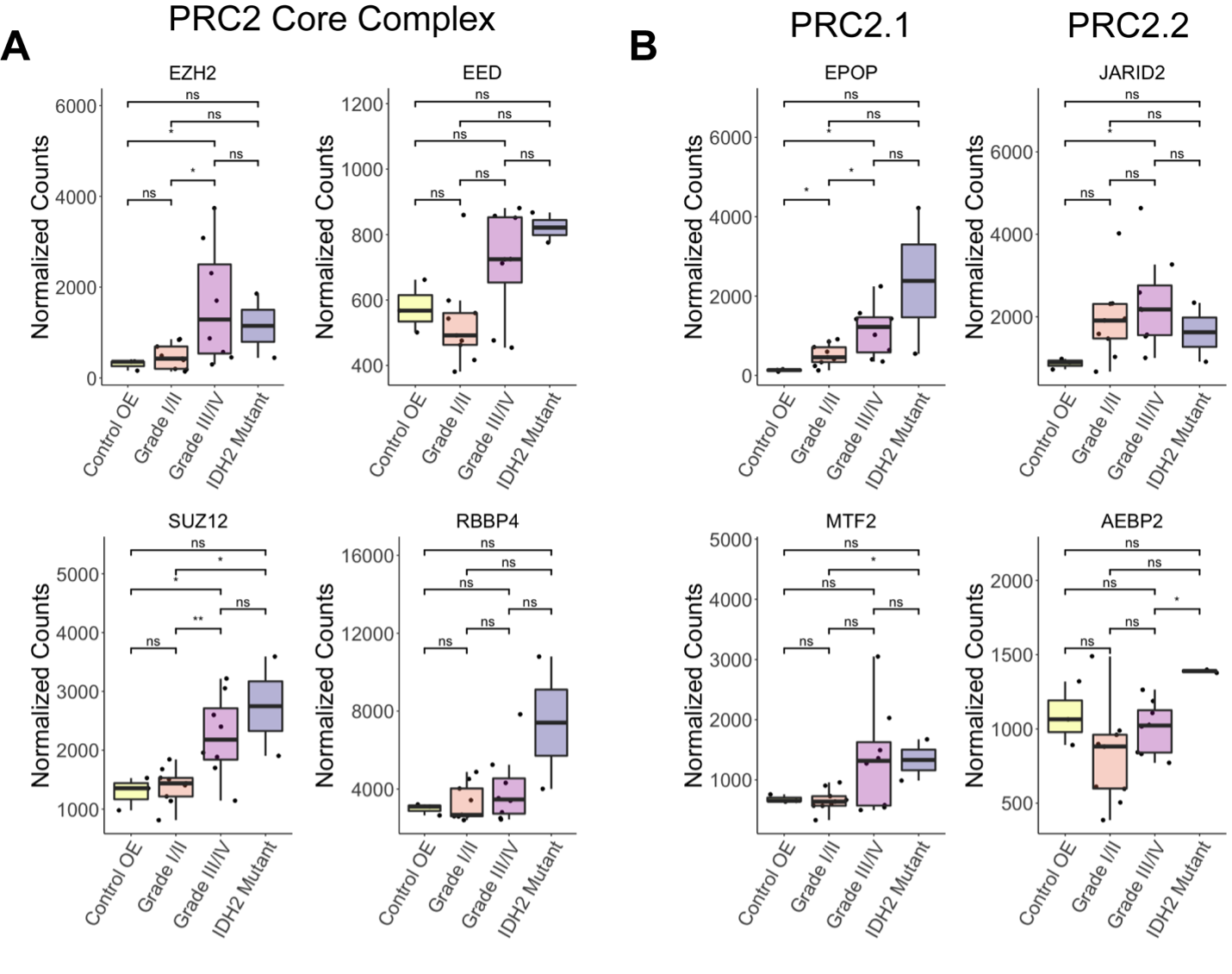

Supplement: Figure S3 — Identical RNA expression plots to those shown in Fig. 4 A and B, but with IDH2 mutant ONB separated out from wild type tumors. [file crc-23-0013-s04.png]

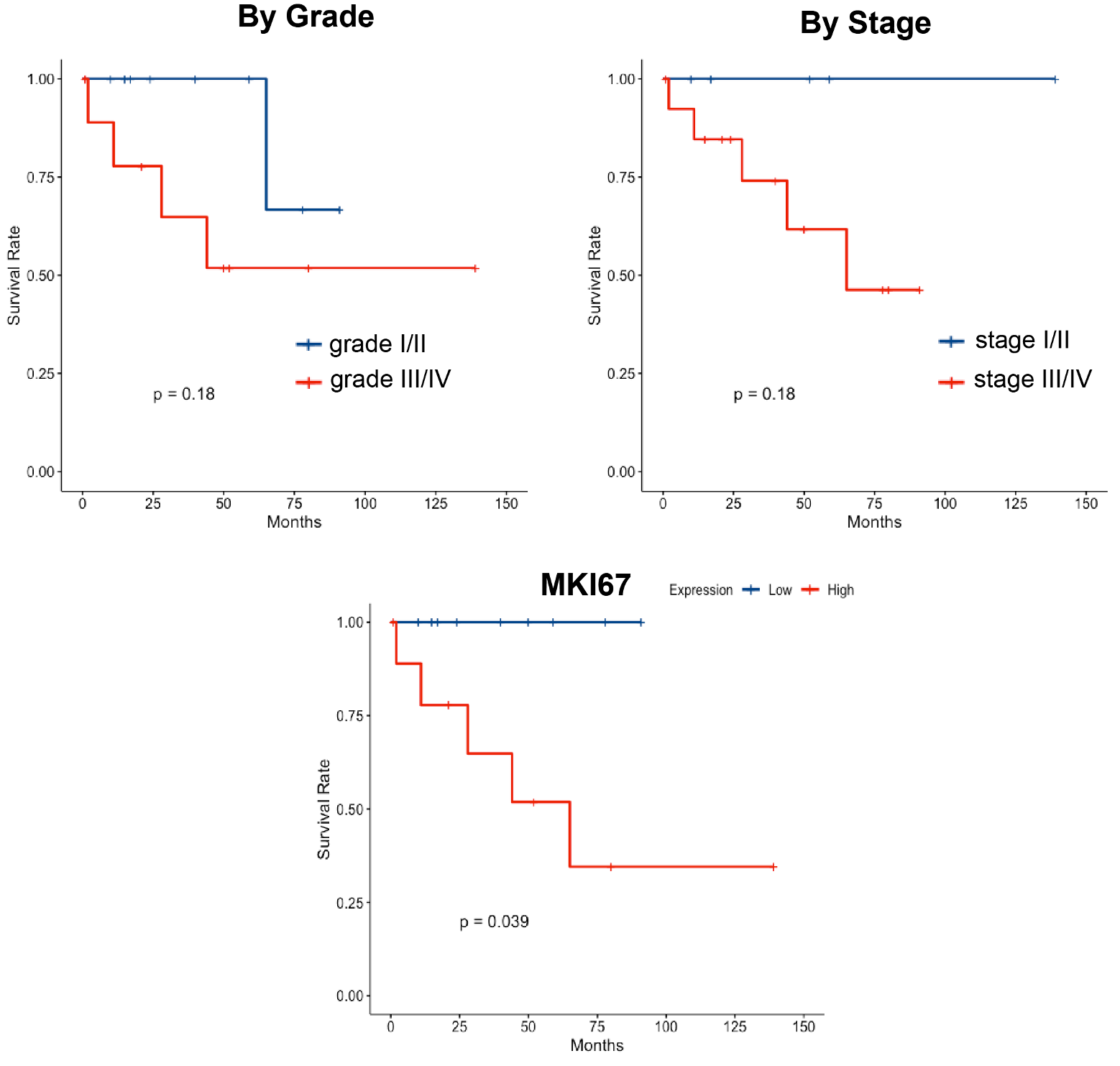

Supplement: Figure S4 — Additional Kaplan-Meier survival curves based on Hyams Grade, Dulguerov T-stage, and MKI67 gene expression. Log-rank test used to determine statistical significance. [file crc-23-0013-s05.png]
